# Regulations Governing Medicines for Maternal and Neonatal Health: A Landscape Assessment

**DOI:** 10.1007/s43441-023-00593-3

**Published:** 2023-12-17

**Authors:** Amalia Alexe, Anju Garg, Birgit Kovacs, Nadezda Abramova, Olatayo Apara, Osa Eisele, Maria Fernanda Scantamburlo Fernandes, Leesha Balramsingh-Harry, Keele Wurst, David Lewis

**Affiliations:** 1 Novartis, Rue de La Tour de L`Ile 4, 1204 Geneva, Switzerland; 2 Sanofi, Bridgewater, USA; 3 Boehringer Ingelheim Pharmaceuticals, Danbury, USA; 4 Merck Healthcare KGaA, Frankfort, Germany; 5 Merck & Co., Inc., Philadelphia, USA; 6 Amgen Inc., Los Angeles, USA; 7 Eli Lilly, Indianapolis, USA; 8 Hoffman LaRoche Ltd., Mississauga, Canada; 9 GlaxoSmithKline, Raleigh, USA

**Keywords:** Pharmacovigilance regulations, Patient safety, Pregnancy, Breastfeeding

## Abstract

Limited evidence related to the safety or efficacy of medicines in pregnancy and during breastfeeding is available to inform patients and healthcare professionals. Understanding the current regulatory landscape in the clinical trial and postmarketing settings is critical to facilitate the development of applicable processes and tools for studying medicine use during pregnancy and breastfeeding and comply with health authority expectations. This review summarizes key findings from a landscape assessment of regulations, guidelines, and guidance on the use of medicines in pregnancy and breastfeeding issued by health authorities in various territories (including the Americas, Europe, Africa, and Asia Pacific) and outlines relevant initiatives undertaken by health authorities, academic institutions, industry consortia, and public–private organizations. While global pharmacovigilance legislation regarding medication use during pregnancy and breastfeeding exists and continues to evolve, the landscape assessment revealed that there is a lack of global legislative harmonization in both the clinical trial and postmarketing surveillance settings and regulatory gaps still exist in many countries/regions. Despite ongoing efforts from health authorities and public and private organizations, intensive efforts for legislation harmonization and stakeholder collaboration are required to improve the current environment of medication safety in pregnancy and breastfeeding.

## Introduction

Each year, approximately, 140 million births occur worldwide [1]. Yet while 44% to 99% of pregnant women take medications during their pregnancy, pregnant women remain an understudied population [2]. For example, in the last 40 years, only one medicinal product (atosiban) was developed and approved for use during pregnancy in the United Kingdom (UK) to halt premature labor, and only five other medications (for various indications) are currently licensed for non-obstetric use during pregnancy [3]. Thus, limited evidence related to the safety or efficacy of medicines in pregnancy is available to inform patients and healthcare professionals on the benefit/risk balance to the mother and fetus. Consequently, some women with chronic diseases are non-adherent to maintenance treatment during pregnancy due to a fear that their medications are unsafe for their unborn child [4].

The topic of medication use during pregnancy and while breastfeeding continues to evolve as the regulatory environment includes both established standards, such as the International Council for Harmonisation of Technical Requirements for Pharmaceuticals for Human Use (ICH) Guidelines [5], as well as newly emerging standards, including the soon to be effective European Medicines Agency (EMA) Good Pharmacovigilance Practices (GVP) Chapter P.III [6]. Globally, various initiatives exist to improve this knowledge gap, with diverse efforts spanning health authorities, academic institutions, industry consortia, and public–private organizations to meet this challenge (e.g., Innovative Medicines Initiative [IMI] ConcePTION, Association of the British Pharmaceutical Industry [ABPI] Maternal Health Project Group, United States [US] Task Force on Research Specific to Pregnant Women and Lactating Women [PRGLAC]) [7–9]. Moreover, the conventional attitude to protect pregnant women from participation in clinical trials has evolved to carefully consider the inclusion of pregnant women based on clinical need and ethical considerations [10].

Understanding the current regulatory landscape in the clinical trial and postmarketing settings is imperative to facilitate the development of applicable processes and tools for studying medicine use during pregnancy and breastfeeding and to comply with health authority expectations. TransCelerate BioPharma is a non-profit organization with more than 20 biopharmaceutical member companies that aim to streamline and accelerate the research and development of new therapies around the world. To meet the need for a regulatory landscape assessment on this topic, TransCelerate formed the Pharmacovigilance Pregnancy and Breastfeeding Topic Team to map existing global regulations,guidelines, and guidance on the use of medicines in pregnancy and breastfeeding, with the ultimate goal of using this understanding to propose solutions with a patient-centric approach [11]. This review summarizes key findings from the landscape assessment of regulations, guidelines, and guidance concerning the use of medicine during pregnancy and breastfeeding issued by health authorities in various countries. This paper also outlines relevant initiatives undertaken by health authorities, academic institutions, industry consortia, and public–private organizations.

## Methods

**Figure 1:**
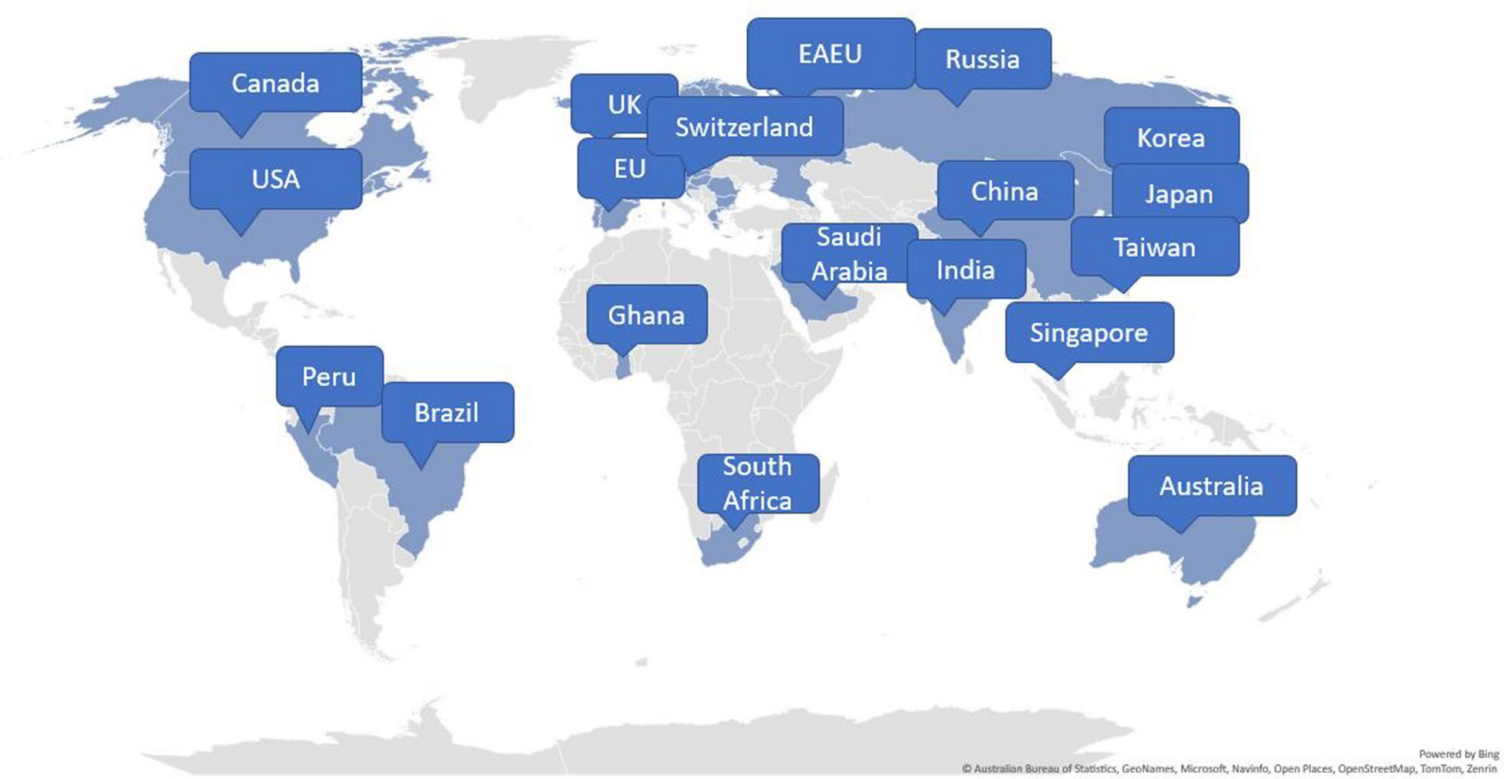
Map of the countries and regions included in the landscape assessment. EAEU, Eurasian Economic Union; EU, European Union; UK, United Kingdom; USA, United States of America.

For the landscape assessment, an in-depth search and review of global regulatory guidance and legislations were conducted following the “four-eyes principle” (reviewed by two team members). Findings were consolidated following an independent review. Territories in scope included the Americas, Europe, Africa, and Asia Pacific (Fig. 1). In and out of scope topics for the reviewed regulations, guidelines, and guidance covering clinical trial and postmarketing settings are outlined in Table 1. The ICH standards and the Council for International Organizations of Medical Sciences (CIOMS) guidelines served as benchmarks for national safety regulations, guidelines, and guidance [5, 12]. To provide a comprehensive evaluation, initiatives across private consortia, health authorities, and academia have also been included in this landscape assessment. The landscape assessment was conducted based on information that was available as of March 2022. Further changes in the regulatory landscape after March 2022 are not comprehensively reflected in this review.

**Table 1:** In and out of scope topics for the regulations, guidelines, and guidance landscape assessment

In scope	Out of scope
Maternal, breastfeeding, and paternal exposure Case reports Aggregate reports Enhanced pharmacovigilance Signal detection Risk management Inclusion/exclusion criteria and enrollment in clinical trials Postmarketing surveillance Pregnancy registries Labeling regulations	Preclinical safety regulations Regulations on contraception Medical device regulations Submission requirements for clinical trial case reports

The content in this Paper is provided for informational purposes only and should not be construed as conveying legal advice. Any party using these materials to determine the regulatory landscape across jurisdictions for purposes of drug development, drug approval, or patients safety or any other purposes bears sole and complete responsibility for determining what laws, regulations, and guidances apply to its conduct and operations in each relevant jurisdiction and complying with (including how best to comply with) all applicable laws and regulations in all relevant jurisdictions. The views and opinions expressed herein are those of the authors; they do not necessarily reflect those of their affiliated companies.

## Results

### Regulations, Guidelines, and Guidance

Key results from the TransCelerate landscape assessment are summarized below [13]. These results provide information on which topics in the clinical trials and postmarketing settings (Table 1) have regulations, guidelines, and guidance from ICH, CIOMS, or at a national/regional level.

The following should be noted regarding regulations, guidelines, and guidance at a national/regional level:

- ICH guidelines are adopted by Brazil, Canada, China, the European Union (EU), Japan, Korea, Saudi Arabia, Singapore, Switzerland, the UK (which became a member in June 2022), and the US [14]
- Eurasian Economic Union (EAEU) regulations, guidelines, and guidance apply to Armenia, Belarus, Kazakhstan, Kyrgyzstan, and Russia [15]

Detailed specifics of the reviewed regulations or guidances can be found in the complete TransCelerate landscape assessment output [13].

### Clinical Trials Regulations, Guidelines, and Guidance

Regulations, guidelines, and guidance specific to the clinical trial setting are summarized in Tables 2 and 3 (topics: enrollment in studies, follow-up, lactation studies) and in Table 4 (topics: case reports, aggregate reports, risk management). When available, requirements from ICH and CIOMS regulations for each topic are presented in the summary tables alongside country-specific legislation (denoted by filled in boxes). ICH member status of the countries/regions included in the landscape assessment (Fig. 1) is also noted, as membership indicates adoption of ICH guidelines in that country.

**Table 2:**
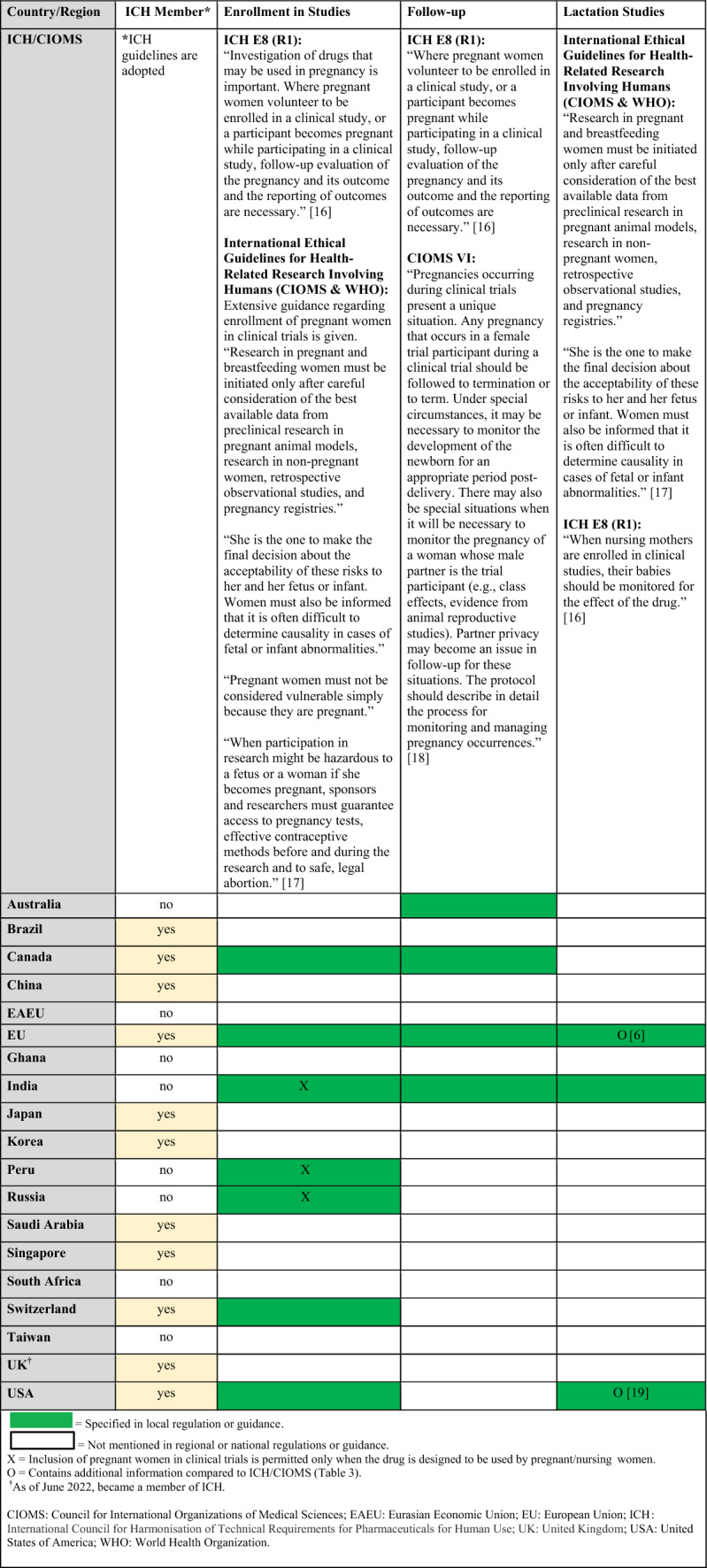
Clinical Trials: Summary of regulations, guidelines, and guidance by country and topic (enrollment in studies, follow-up, lactation studies) and comparison to ICH/CIOMS guidelines

**Table 3:** Clinical Trials: Additional regional guidances on lactation studies beyond ICH/CIOMS guidelines

Country/region	Lactation studies
EU	EMA Guideline of GVP Chapter P.III.B.4.3 Clinical Lactation Studies: “In cases where no human data are available on the extent of medicine transfer into breast milk, where use by breastfeeding women is expected to be common, and based on the medicinal product’s pharmacological properties, it is considered plausible that there is a risk to breastfed infants, a PK study amongst breastfeeding women should be considered. This is expected to be the case when a medicinal product is commonly used by women of reproductive age (e.g., antidepressants, anti-infectives, diabetes medications, pain medications) or when there is evidence of use or anticipated use of the medicinal product by lactating women.” “Medicine concentration levels in breast milk samples should be measured and a relative infant dose calculated, to obtain information for supporting the risk assessment and provision of advice on timing of medicine intake relative to breastfeeding where this may be feasible (e.g., for short-term or single-dose treatments). Moreover, data on the effect of the medicine on milk production or composition should be collected, if potentially clinically relevant.” “In the case of a medicine highly used in women who could breastfeed, with an unknown potential for serious adverse reactions in breastfed children, establishing safety information in the post-authorisation phase should be considered as an important source of information. This may include the clinical follow-up of breastfed children whose mothers are treated with a specific medicine. Pregnancy registries in which newborns are further observed could include the collection of information on breastfeeding to allow a comparison of a group of breastfed children to those not breastfed and those breastfed in mothers who are not treated with the product of interest. In case a medicine is used during breastfeeding and questions arise regarding a potential long-term impact on child’s growth, neurodevelopment, or other adverse events with a prolonged latency, it should be considered to carry out long-term follow-up in those children.” [6]
USA	FDA Guidance for Industry—Clinical Lactation Studies—Considerations for Study Design: Detailed design considerations for lactation studies are provided, including sample collection, pharmacokinetic and pharmacodynamic considerations. [19]

*CIOMS* council for international organizations of medical sciences, *EMA* European medicines agency, *EU* European union, *FDA* food and drug administration, *GVP* good pharmacovigilance practices, *ICH* international council for harmonisation of technical requirements for pharmaceuticals for human use, *USA* United States of America

**Table 4:**
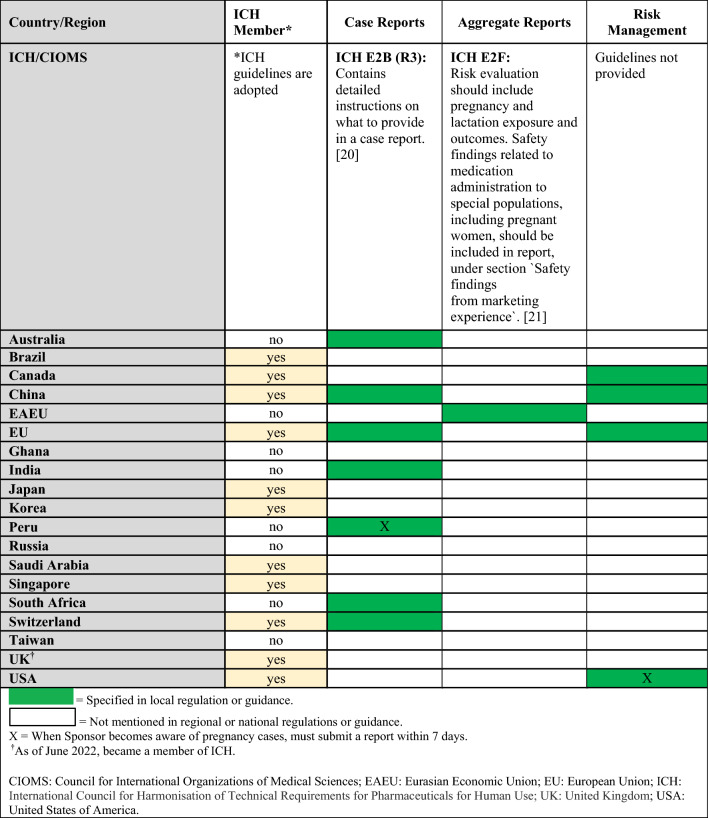
Clinical Trials: Summary of regulations, guidelines, and guidance by country and topic (case reports, aggregate reports, risk management) and comparison to ICH/CIOMS guidelines

Regulatory gaps (denoted as blanks in the tables) as well as inconsistencies among territories were observed (Tables 2, 3, and 4) [6, 16–21]. Additionally, ICH regulation lacks granularity in the clinical trials setting and enrollment regulations vary among countries (Table 2). Risk management measures are generally focused on contraception, which was an out of scope topic for the landscape assessment (Tables 1 and 4).

### Postmarketing Surveillance Regulations and Guidelines

Regulations, guidelines, and guidance specific to the postmarketing surveillance setting are summarized in Table 5 (topics: case reports, follow-up reports), Tables 6 and 7 (topics: postmarketing studies, pregnancy registries), Tables 8 and 9 (topics: risk assessment and planning, signal detection, aggregate reports), and in Table 10 (topic: labeling). When available, requirements from ICH and/or CIOMS regulations for each topic are presented in the summary tables alongside country-specific legislation; filled in boxes denote available regulation/guidance, whereas blanks in the tables denote regulatory gaps. When relevant, the tables also specify which countries/regions follow ICH guidelines (ICH member status).

**Table 5:**
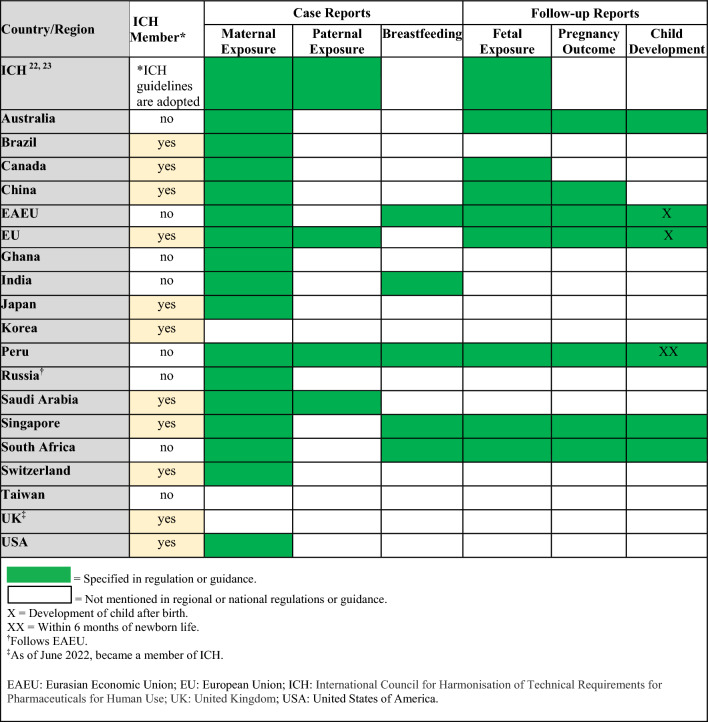
Postmarketing surveillance: summary of ICH and regional/local regulations, guidelines, and guidance for specific types of case reports and follow-up reports

**Table 6:**
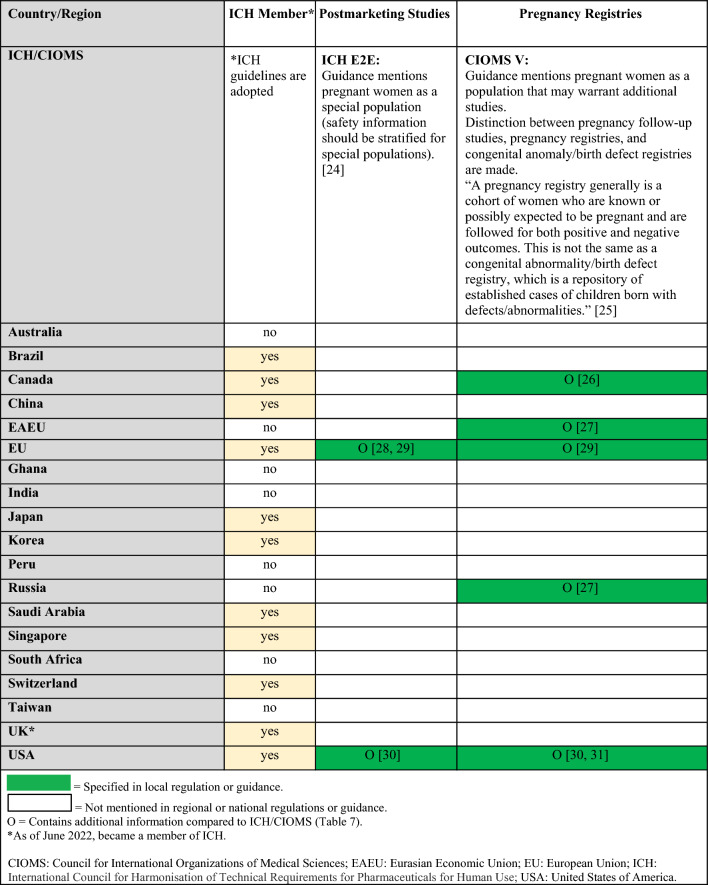
Postmarketing surveillance: summary of regulations, guidelines, and guidance by country and topic (postmarketing studies, pregnancy registries) and comparison to ICH/CIOMS guidelines

**Table 7:**
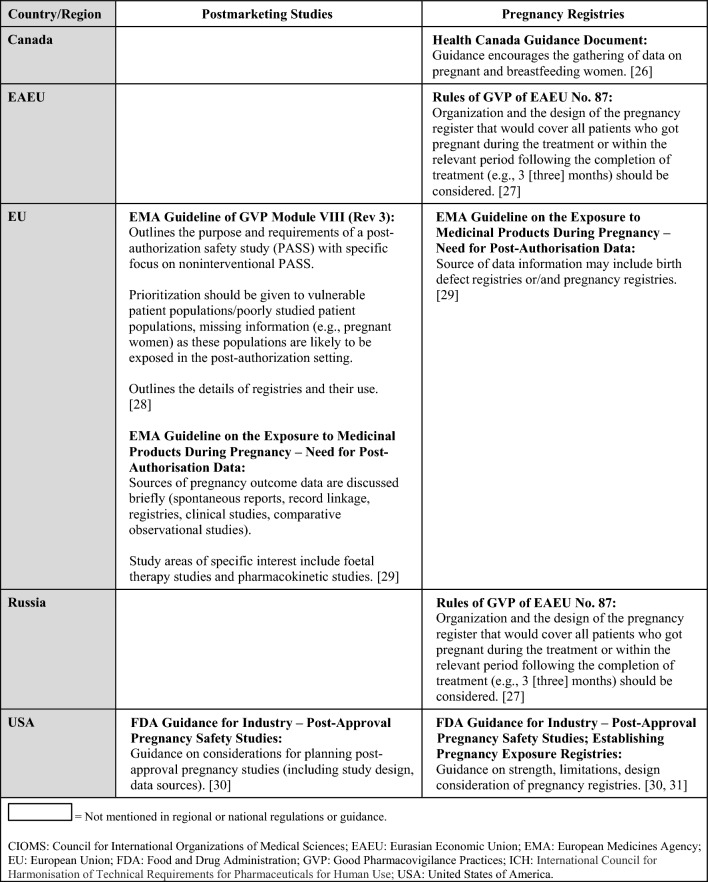
Postmarketing surveillance: additional regional guidances on postmarketing studies and pregnancy registries beyond ICH/CIOMS guidelines

**Table 8:**
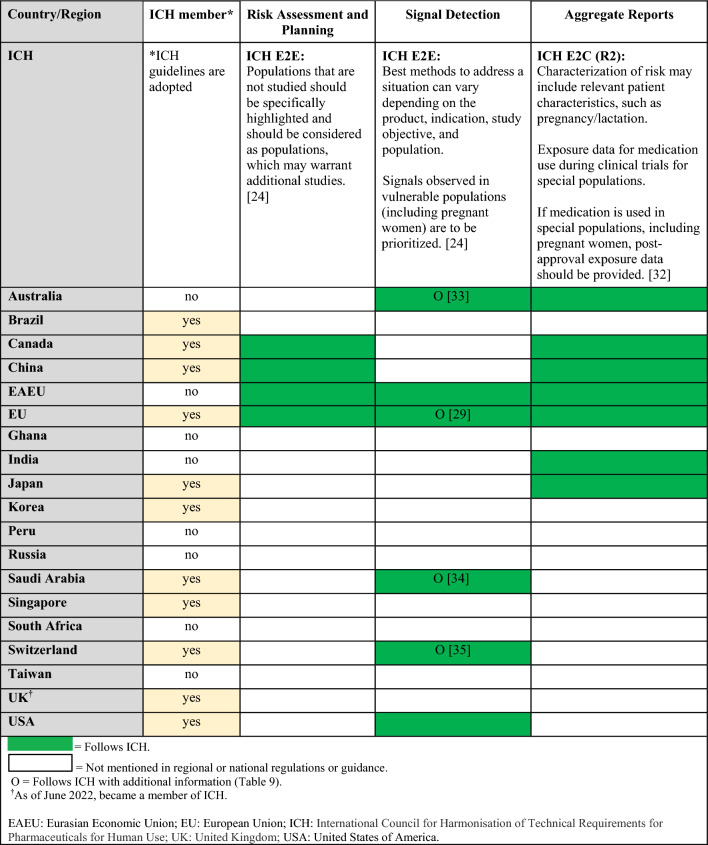
Postmarketing surveillance: summary of regulations, guidelines, and guidance by country and topic (risk assessment and planning, signal detection, aggregate reports) and comparison to ICH guidelines

**Table 9:** Postmarketing surveillance: additional regional guidances on signal detection beyond ICH guidelines

Country/region	Signal detection
Australia	TGA Pharmacovigilance Responsibilities of Medicine Sponsors: Report signal of a possible teratogenic effect (cluster of cases) as a significant safety issue [33]
EU	EMA Guideline on the Exposure to Medicinal Products During Pregnancy—Need for Post-Authorisation Data: Additional guidance on how to perform signal detection for pregnancy cases [29]
Saudi Arabia	SFDA Guideline on GVP (v3.1): Immediately notify about potential signals of teratogenicity [34]
Switzerland	Swissmedic: Additional guidance regarding potential/identified signals of teratogenicity [35]

*EMA* European medicines agency, *EU* European union, *GVP* good pharmacovigilance practices, *ICH* international council for harmonisation of technical requirements for pharmaceuticals for human use, *SFDA* Saudi food and drug authority, *TGA* therapeutic goods administration

**Table 10:**
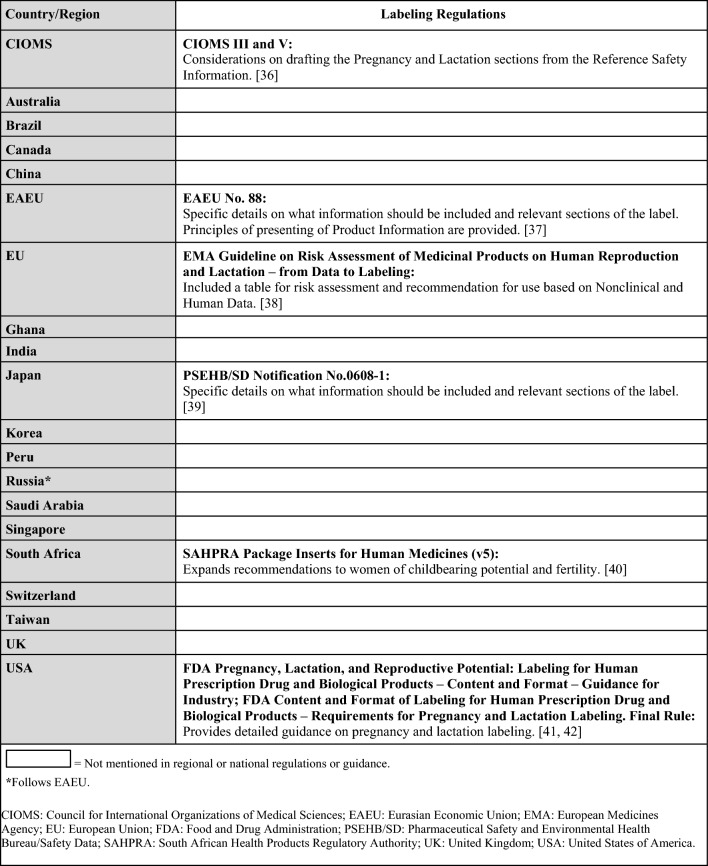
Postmarketing surveillance: summary of regulations on labeling by country and CIOMS guidelines

With regard to different types of case reports, ICH guidelines mention maternal exposure and paternal exposure to a drug but do not address drug exposure via breastfeeding (Table 5) [22, 23]. Nonetheless, national/regional regulations or guidances address these types of case reports with exception of Korea, Taiwan, and the UK (Table 5). With regard to different types of follow-up reports, there are no ICH guidelines for follow-up reports on pregnancy outcomes or child development but ICH does instruct to follow-up all reports of possible fetal exposure to the medical product and to consider product half-life [22, 23]. Conversely, some national/regional guidances specifically require follow-up on pregnancy outcomes and/or monitoring of child development (Table 5).

There are ICH or CIOMS guidelines, as well as regional regulations or guidances, for postmarketing studies, pregnancy registries, risk assessment and planning, signal detection, and aggregate reports [24–35]. Tables 6 and 8 summarize which countries/regions have regulations or guidances for these topics. If national/regional regulations or guidance exist, they follow ICH, although some may have additional details for these topics (e.g., organization or design of registries, reporting timeline to health authorities for a signal, additional guidance for signal detection) that are described in Tables 7 and 9 [26–31, 33–35].

Lastly, while CIOMS has recommendations on labeling, several countries/regions also have labeling regulations or guidances that provide more detailed information compared to CIOMS (Table 10) [36–42].

## Pregnancy- and Breastfeeding-Related Initiatives

**Figure 2:**
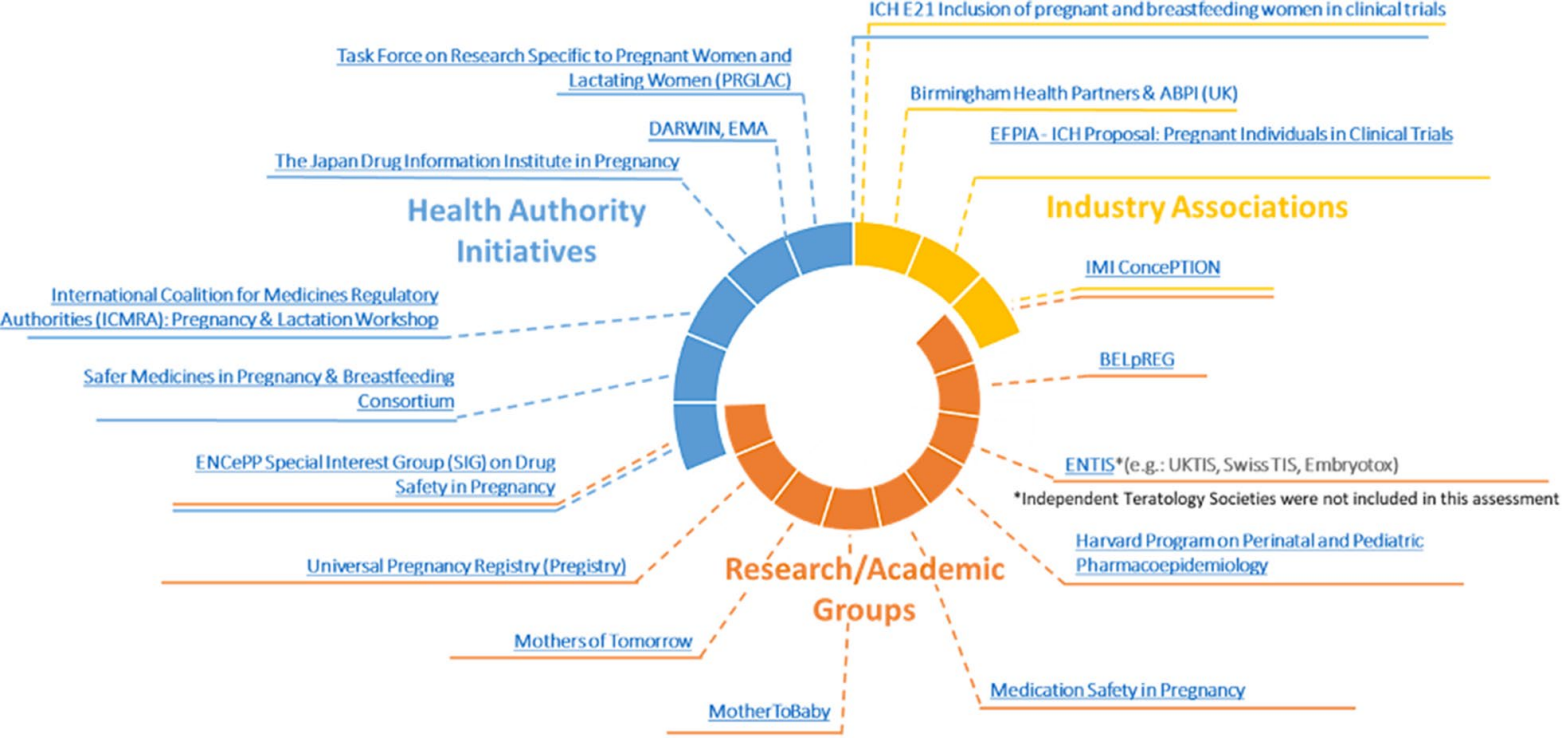
Pregnancy and breastfeeding initiatives from health authorities, industry associations, and research/academic groups. Relevant initiatives include the Association of the British Pharmaceutical Industry (ABPI) Maternal Health Project Group [8], the BELgian interdisciplinary initiative to enhance pregnancy related data REGistration and research on medication use (BELpREG) [43], the European Federation of Pharmaceutical Industries and Associations (EFPIA)—International Council for Harmonisation of Technical Requirements for Pharmaceuticals for Human Use (ICH) Proposal on Pregnant Individuals in Clinical Trials [44], the European Network of Centres for Pharmacoepidemiology and Pharmacovigilance (ENCePP) Special Interest Group on Drug Safety and Pregnancy [45], the European Network of Teratology Information Services (ENTIS) [46], the Innovative Medicines Initiative (IMI) ConcePTION [7], the United States Task Force on Research Specific to Pregnant Women and Lactating Women [9], the Japan Drug Information Institute in Pregnancy [47], the International Coalition for Medicines Regulatory Authorities (ICMRA) Pregnancy and Lactation Workshop [48], the United Kingdom (UK) Safer Medicines in Pregnancy and Breastfeeding Consortium [49], the Universal Pregnancy Registry (Pregistry) [50], Mothers of Tomorrow [51], MotherToBaby [52], Medication Safety in Pregnancy [53], and the Harvard Program on Perinatal and Pediatric Pharmacoepidemiology [54].

Related initiatives conducted by health authorities, industry associations, and research/academic groups focus on a wide array of objectives, including pregnancy/breastfeeding data, policy information, clinical trials, and patient communication. Most initiatives are led by research/academic groups or health authorities and primarily focus on data collection or provision of information (Fig. 2) [7–9, 43–54]. Details on specific initiatives can be found in the complete TransCelerate landscape assessment output [13]; this output should not be considered a comprehensive list of initiatives, but an overview of main associations that aim to improve understanding of medication efficacy and safety in pregnancy and breastfeeding.

## Discussion

Limited evidence related to the safety or efficacy of medicines in pregnancy is available to inform patients and healthcare professionals on the benefit/risk balance to the mother and fetus. While the majority of pregnant individuals take at least one medication during their pregnancy, only a few medications were developed to be used by pregnant people [2, 3]. Moreover, less than 25% of the medications available on the market present concrete information regarding risks during pregnancy in the product label. There is a dire need to understand and overcome the scientific, legislative, legal, and ethical challenges preventing the development of safe and effective medicinal products for use during pregnancy and while breastfeeding.

As a first step toward meeting this challenge, this regulatory landscape assessment, developed by experts in pharmacovigilance and/or maternal and fetal health, focused on regulatory challenges and represents an overview of current safety legislation for pregnancy and breastfeeding [13]. Based on information that was available as of March 2022, globally, pharmacovigilance legislation regarding medication use during pregnancy and breastfeeding exists (e.g., ICH guidelines, CIOMS recommendations, national legislations) and continues to evolve. For example, the ICH E21 Working Group on Inclusion of Pregnant and Breastfeeding Individuals in Clinical Trials was formed in Q4 2022 and the EMA GVP Chapter P.III pregnancy legislation is expected to be launched in Q3 2023 [6, 55].

However, despite ongoing efforts from health authorities and public and private organizations (e.g., EU IMI ConcePTION, US PRGLAC, national and global teratology centers), the landscape assessment revealed that there is currently a lack of global legislative harmonization in both the clinical trial and postmarketing surveillance settings [13]. While ICH/CIOMS regulations include general provisions on safety in pregnancy and breastfeeding, more details would be required to support development in this area [16–18, 20–25, 32, 36].

Additionally, while several health authorities have made immense progress by providing detailed recommendations in their respective territories (Tables 3, 7, 9 and 10), regulatory gaps still exist in many countries/regions that were included in the landscape assessment (Fig. 1) [6, 19, 26–31, 33–42]. In particular, significant regulatory gaps exist in the clinical trials setting [e.g., lack of regulations or granularity in the regulations for pregnancy or lactation studies (Tables 2 and 4)], whereas postmarketing surveillance legislation is generally further developed (Tables 5, 6, 8, and 10). In some instances, local regulations are more specific regarding signal management than ICH guidelines [e.g., focus on fetotoxicity in Australia, Saudi Arabia, and Switzerland (Tables 8 and 9)] [33–35].

Of note, no end-to-end product development guideline exists for medications to be used by pregnant women. Moreover, where national legislation on related topics exists, global inconsistencies among national requirements in the clinical trials setting were observed. For example, requirements for enrolling pregnant or nursing women into clinical trials vary; in India, Peru, and Russia, enrolling pregnant women into clinical trials is only permitted if the medication is designed specifically for use in this population, while in Canada, EU, Switzerland, and the US, enrollment is permitted after careful benefit/risk assessment, including the mother and the fetus (Table 2). In the postmarketing surveillance setting, requirements for post-authorization study design differ between the EU and US (Table 7). Recommendations for case collection after exposure to medication during breastfeeding or related to longer term follow-up vary as well. These aspects lead to a lack of clarity, uncertainty, establishment of complex pharmacovigilance processes, and delays when it comes to the much-needed product development for this population.

There is an acute need to harmonize global legislation for medication safety in pregnancy and breastfeeding and to provide end-to-end product development guidance for medications to be used in this population. While no investigational plan has been proposed or is required by health authorities in this area, discussions to develop a “maternal” or an “obstetric” investigational plan are currently ongoing in several territories. In 2021, the International Coalition of Medicines Regulatory Authorities (ICMRA) workshop (attended by the EMA and US Food and Drug Administration [FDA] representatives) called for the development of a maternal investigational plan, to be proposed by sponsors, outlining how these populations will be studied in the product development [48]. Similarly, discussions regarding an obstetric investigational plan, based on learnings from the successful pediatric investigational plans, are occurring in the UK [49].

Based on findings of the landscape assessment, the TransCelerate Pharmacovigilance Pregnancy and Breastfeeding Topic Team has developed a openly available toolkit (called `Points to Consider Concerning the Use of Medicines in Pregnancy throughout the Product Lifecycle`) that aims to provide a holistic view of pregnancy considerations across the lifespan of the drug and aid researchers to optimize their compliance with regulatory authority expectations [56].

## Conclusions

While global pharmacovigilance legislation regarding medication use during pregnancy and breastfeeding exists and continues to evolve, intensive efforts for legislation harmonization and stakeholder collaboration are required to improve the current environment of medication safety in pregnancy and breastfeeding. Sponsors, marketing authorization holders, researchers, healthcare professionals, and patients must work together to enhance medicinal product development, data collection, and transparent risk communication to ultimately improve maternal and fetal health outcomes following medication exposure for the generations to come.

## Data Availability

The data that support the findings of this paper are openly available on the TransCelerate platform at Regulatory Landscape Assessment.
